# A mixed-methods strategy to analyse the dynamics of safety violations of the construction workers in Hong Kong

**DOI:** 10.1016/j.mex.2025.103687

**Published:** 2025-10-27

**Authors:** Wing Chi Tsang, Shoeb Ahmed Memon, Steve Rowlinson

**Affiliations:** a School of Science and Technology, Hong Kong Metropolitan University, Hong Kong SAR, China; b Faculty of Society and Design, Bond University, 14 University Drive, Robina, Gold Coast, QLD 4226, Australia; c Department of Real Estate and Construction, Faculty of Architecture, The University of Hong Kong, Pokfulam, Hong Kong SAR, China

**Keywords:** Construction safety, Theory of planned behaviour, High reliability organising, Safety violations

## Abstract

There is a lack of research specifically addressing safety violations among construction workers in Hong Kong. This study, therefore, aims to address the research gap and provide insight into the causes of safety violations. This study employs a mixed-methods approach to achieve research objectives and leverage the benefits of each method.

Using a Theory of Planned Behaviour framework and High Reliability Organising (HRO), this research adopted a questionnaire survey to test the relationships among the constructs, which obtained 365 valid responses.

Subsequently, 37 semi-structured interviews were conducted to gain insight into workers’ perspectives.

The research results confirmed reliability and validity of the employed methods. Intention significantly affects the occurrence of safety violations. Among cognitive determinants of safety violations, two factors have a notable influence on intention, with Perceived Behavioural Control (PBC) being the most critical factor. HRO, as the distal factor that impacts cognitive determinants of safety violations, initiates a transformative approach to construction safety management that emphasises continuous reflection and improvement. Practical strategies are recommended to target PBC and attitude to improve workers’ intentions. It is also recommended that training be designed to cater to contextual features of different work groups, such as young and elderly workers.

## Graphical abstract

**Figure:**
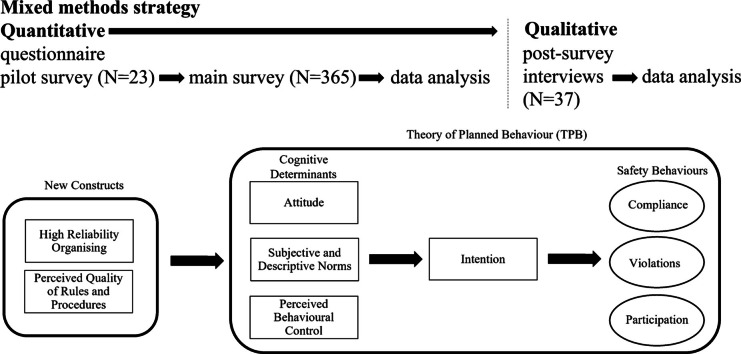
Mixed methods strategy adopted in this study.

Specifications table

**Table:** 

**Subject area**	*Engineering*
**More specific subject area**	*Construction safety management*
**Name of your method**	*Mixed-methods strategy: quantitative and qualitative analysis*
**Name and reference of original method**	I. Ajzen, University of Massachusetts Amherst, Constructing a Theory of Planned Behavior Questionnaire. http://people.umass.edu/aizen/pdf/tpb.measurement.pdf, 2015 (accessed 21 November 2015).
**Resource availability**	Resources referred to in this article: • Statistical Package for the Social Sciences (SPSS) (https://www.ibm.com/spss) • Analysis of Moment Structures (AMOS) (https://www.ibm.com/products/structural-equation-modeling-sem) • NVivo (https://lumivero.com/products/nvivo/)

## Introduction

While awareness of safety in construction is growing, the accident rates have recently plateaued. This trend is observed worldwide, including in Hong Kong, as shown by recent statistics from different nations [1]. Additionally, the construction sector in Hong Kong is grappling with the challenge of an ageing workforce. Even though older workers bring experience and deliver higher-quality workmanship compared to their younger counterparts, they tend to exhibit poorer safety practices and fitness levels, and they often resist change [2]. This is why unsafe behaviours of construction workers are identified to contribute significantly to construction accidents [1].

Due to the scarcity of pertinent studies, this research aims to offer insights into the safety violations. Distinct from prior investigations, this research employs the High Reliability Organising (HRO) mindset that highlights mindfulness and reflection of construction organisations [3]. Therefore, this research investigates factors leading to safety violations. Further, it validates a comprehensive framework for explaining safety violations of construction workers. The framework is further strengthened with in-depth interviews.

### Literature review

#### Safety violations

Through the lens of socio-technical system thinking, safety violations of construction workers are influenced by their interconnected work relationships as well as the processes and methods employed [4], emphasising the intricate and dynamic nature of construction projects. More precisely, these violations pertain to adherence to safety rules and procedures, given that the occurrence of safety violations is tied to the existence of these rules [5]. Conversely, safety compliance is described as an overall expression of safety behaviour in the work of Hayes et al. [6].

### Theory of planned behaviour (TPB)

The Theory of Planned Behaviour (TPB) was developed to explain human behaviour in a variety of contexts [7]. Where theory suggests intention as a critical predictor of human actions, which denotes individuals’ willingness for an action. Intention is influenced by cognitive factors: attitude, norms (subjective and descriptive), and perceived behaviour control. Attitude reflects the value of that behaviour, whereas norms pertain to how others perceive that action (subjective norms) and their likelihood of participating in it (descriptive norms). The original TPB focuses solely on subjective norms. Recent research conducted by Fugas, Silva, and Meliá [8] suggests that it is beneficial to analyse coworkers' and supervisors' norms separately. Perceived behavioural control relates to individuals' beliefs in their capacity to act. Although the TPB has been investigated in previous studies, it has not been well-adapted to understand safety violations among construction workers in Hong Kong.

Based on the assumption of sufficiency, attitude, norms, and PBC should accurately predict intention. General traits such as personality are considered contextual factors and are believed to mediate relationships outlined in the TPB framework. Research suggests that any additional predictor should be integrated with caution [9]. Based on the literature review and the unique characteristics of Hong Kong’s construction industry, two key concepts emerge as essential for understanding the current landscape: (1) the perceived quality of safety rules and procedures, and (2) High Reliability Organising (HRO).

### Quality of safety rules and procedures

The “perceived quality of safety rules and procedures” is central to the occurrence of safety violations. These rules may not always be effective or appropriately enforced in every situation [10]. Cox and Cheyne [11] postulate that workers’ perceptions of safety rules and procedures influence overall safety. Effective rules and procedures are aimed at achieving the right objectives, being appropriately applied, and being clearly presented to discourage employees from breaching them [12]. It is recommended that rules be tailored to the circumstances and the capabilities of individuals using them [13]. Safety violations are more likely to happen when workers view the quality of rules and procedures as subpar.

### High reliability organising (HRO)

According to Harvey et al. [14], companies could enhance their resilience by integrating employee-level components of the “Adaptive” safety era. HRO refers to the capability of an organisation (construction in this case) to anticipate and navigate in situations when safety incidents occur [3]. Harvey et al. [15] highlight both the challenges and opportunities associated with implementing HRO principles and resilience engineering within the construction sector. It advocates for their integration into the evolving paradigm of adaptive safety. Accordingly, HRO can be conceptualised as a distal, organisational factor that significantly influences safety violations of construction workers.

## Methodology

Research methodology is influenced by the research problem [16]. For example, Alper and Karsh [17] suggest employing various methods to comprehend safety violations, because while counting them is straightforward, analysing their underlying causes via observations is more challenging. While a vast number of quantitative studies on the TPB have utilised questionnaires, a growing number of studies have argued in favour of qualitative approaches as a means of better exploring the phenomenon. It is because the questionnaires do not illuminate the interconnections of the concepts with the conditions and context from the behaving person’s perspective [18]. Studies may rely too heavily on existing survey instruments, without reflecting on the complex system and inherently interpretative nature of research [19]. Elosta and Alzubi [20] modify the TPB to explore the leadership and behaviour related to safety among construction workers, recommending that future research utilise mixed methods to enhance comprehension.

To fulfil research objectives, a mixed-methods research strategy was adopted, which adds methodological innovation beyond replication of TPB studies. The reality may not be well-explained because other theory-external influences on behaviour are not considered and are hypothesised to be mediated through the TPB. Consequently, the TPB may become “an empty gesture to tick the box”. TPB may also not consider the actual control, adequate opportunities, and resources for behaviour change [21].

The respective strengths and limitations of research methods influence the choice of mixing. Therefore, the hypotheses for the quantitative part of the research were framed based on the modified TPB model, evaluated through a questionnaire survey. Statistical methods were utilised to investigate the interrelations among the variables. This yields generalised insights into the current safety practices among construction workers in Hong Kong. The quantitative results then inform the qualitative interviews by highlighting the significant causes of safety violations, allowing interviewees to disclose the current phenomenon further and explore the dynamics within construction sites in depth, i.e., how and why a particular issue occurs. The interviewees were invited to comment on the survey results and share their views on safety issues openly.

The qualitative approach allows for the consideration of various factors, levels of impact, and how these factors interact [22], which leads to a more profound comprehension of intricate issues and clarifies connections [23]. Rhodes [24] advocates for utilising a qualitative strategy to challenge and enhance prevailing scientific narratives regarding risk behaviour. The mixed methods complement each other, with the interview findings supporting earlier results and offering a detailed contextual understanding of the ongoing issue of safety violations among construction workers.

### Research model and hypotheses development

**Fig. 1:**
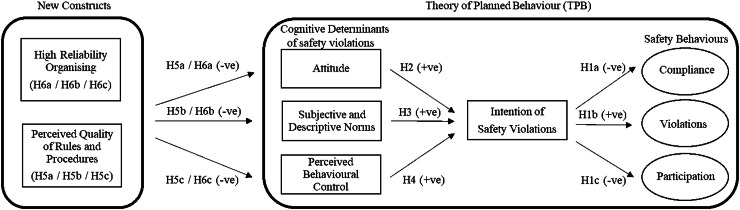
Proposed research model.

Drawing on a comprehensive review of literature and with careful consideration of the contextual nuances and interpretation of findings, the TPB emerges as a robust and distinct framework for investigating safety violations of construction workers in Hong Kong. While it is recognised that the construction sector in Hong Kong includes unique elements, following Ajzen’s principle of sufficiency [9], the initially proposed TPB framework is modified to integrate 1) descriptive norms with subjective norms, (2) perceived quality of safety rules and procedures, and (3) HRO. This study not only explores safety violations but also examines safety compliance and participation among construction workers. Fig. 1 presents the proposed model, and further details can be found in Table 1.

**Table 1:** Details of constructs, hypotheses and associated measurement items [28].

Construct	Description	Hypothesis	Short Description of Measurement Items
Intention of safety violations (I1–I4 a) [29]	Intention can be affected by three proximal factors (attitude, norms, and PBC).	H1a: Intention (of safety violations) has a negative impact on safety compliance. H1b: Intention (of safety violations) has a positive impact on safety violations. H1c: Intention (of safety violations) has a negative impact on safety participation.	- Prepared to take other risks - Prepared to take shortcuts - Must take risks to complete a task - Complete the task in a better way if the procedure is inefficient
Attitude (A1–A2 a) [12,25,26]	Construction workers have a higher intention of safety violations if they are not receptive to following safety rules and procedures.	H2: Attitude has a positive impact on intention.	- It is good to follow rules and procedures - Worthwhile to follow rules and procedures
Subjective and descriptive norms (N1–N6 a) [12,25,26]	Construction workers would have a higher intention when their coworkers and supervisors are less determined for safety and are seen as not consistently adhering to safety rules and procedures.	H3: Norms have a positive impact on intention.	Subjective norms - Supervisors recognise unavoidable deviations from rules - Coworkers and workgroups recognise unavoidable deviations from rules Descriptive norms - Supervisors force individuals to violate rules - Supervisors do not take action against those who break rules - Coworkers and workgroups coerce others into rule-breaking - Coworkers and workgroups use varying standards
PBC (P1–P3 a) [12,25,26]	Evaluates the workers’ view on their ability and resources accessible for adhering to safety rules and procedures.	H4: PBC has a positive impact on intention.	- Working conditions stop me from working to the rules - Find better ways of doing my job - Can finish the job quicker - Lack of adequate resources leads to violations of rules
Perceived quality of safety rules and procedures (Q1–Q12 a) [12]	When workers think that the safety rules and procedures are of higher quality, they have a more negative attitude, norms, and PBC on safety violations.	H5a/H5b/H5c: Perceived quality of safety rules and procedures has a negative effect on attitude (H5a)/norms (H5b)/perceived behavioural control (H5c) of safety violations.	- Rules do not outline the most effective method of working - Timelines provide inadequate time to finish the task - Rules would make jobs less efficient - Rules are hard to apply - Rules frequently refer to other rules - Rules are factually incorrect - Restrictive operating limits stated in rules - No need to follow rules to do the job safely - Rules only protect management - No efficient monitoring procedures - Working by the rules hinders skills - Have rules that are irrelevant to tasks
HRO (H1–H9 a) [3]	Construction workers have a more negative attitude, norms, and PBC of safety violations when they perceive that their organisations have a higher level of HRO characteristics.	H6a/H6b/H6c: HRO has a negative effect on attitude (H6a)/norms (H6b)/PBC (H6c) of safety violations.	- Understand individuals’ abilities and strengths well - Discuss errors made and the lessons that were gained from them - Discuss and know who has specialised skills and knowledge - Discuss alternatives for our everyday work activities - Discuss with coworkers about emerging problems - Make use of the colleagues’ unique skills to resolve a problem - Spend time identifying activities to avoid going wrong - Discuss how we could have prevented errors from happening - Rapidly pool our collective expertise to resolve a crisis
Safety compliance (SC1–SC4 ^b^) [30]	Dependent variable	Refer above	- Work in a safe manner - Use necessary safety equipment - Use the correct safety procedures - Ensure the highest safety standard
Safety violations (SV1–SV3 ^b^) [29]	Dependent variable	Refer above	- Whether approved procedures are followed - Perform a familiar task by referring to the approved documents - “Bend” formal procedures to complete a task on time
Safety participation (SP1–SP3 ^b^) [30]	Dependent variable	Refer above	- Extra effort to improve workplace safety - Help my coworkers under dangerous conditions - Voluntarily carry out tasks to improve safety

a The scale of the measure is as follows: (1) strongly disagree; (2) disagree; (3) sometimes disagree; (4) neither disagree nor agree; (5) sometimes agree; (6) agree; (7) strongly agree. ^b^ The scale of the measure is as follows: (1) never; (2) rarely; (3) occasionally; (4) sometimes; (5) frequently; (6) usually; (7) always.

### Data collection (survey)

The questionnaire adopted a 7-point Likert scale, which required approximately 20–30 min to complete. The scales were designed based on the recommendations from Ajzen [25] and Francis et al. [26]. The measurement of the TPB constructs (attitude, norms, perceived behavioural control, and intention) were developed according to the manual for constructing the TPB questionnaires [26] with reference to the questionnaire wordings of the report “Improving Compliance with Safety Procedures: Reducing Industrial Violations” [12]. Perceived quality of rules and procedures was identified and adopted from the report since it investigates the relevant concept. For HRO, the Mindfulness Organising Scale (MOS) was developed as an economical audit by Weick and Sutcliffe [3], which captures specific behaviours that can be seen in HROs. Each question can be categorised into the HRO principles. The items of safety behaviours (compliances, violations, and participation) were adapted from the existing literature. Demographic variables such as gender, age, race, education, religiosity, marital status, living with children, job nature, working level, and working location are adapted from Barrientos-Gutierrez et al. [27]. All items were reviewed to ensure their relevance to the context of the construction sector in Hong Kong. The measurement items developed in the questionnaire survey are listed in Table 1 below.

The questionnaire was tested in a pilot survey to determine if any modifications were required, and then the main survey was conducted. The participants were asked to confirm whether they had previously filled out the questionnaire to ensure they were distinct from the pilot study. After analysing the quantitative data, post-survey interviews were conducted.

The source language of the questionnaire was English. Since the respondents were local workers whose English proficiency may not be sufficient, the questionnaire was translated into Chinese. Back-translation, that is defined as “render the target language back to the original language without having seen the original text”, was adopted to evaluate the quality of the translation (van de Vijver and Leung, 1997, as cited in Geisinger, 2003, p. 106) [31]. The procedures in Pelletier et al. [32] were referred to. The target language was first translated from the source language, and the preliminary version was evaluated by back-translation with the emphasis on the actual meanings of the items. Several differences were identified and discussed between the back translator and the author. For example, the original term “co-workers” was translated as “同事” in the target language, whereas “colleagues” was used in the back translation. The two terms were distinguished, with the former referring to a working group that implies a closer relationship. Eventually, “同僚” was agreed as the better term for translation. The questionnaires were modified accordingly. The evaluated questionnaire was then pretested for verification by asking three university colleagues of the author to read the questions (both English and Chinese versions) and confirm that they have the same meaning.

After that, the pilot survey tested the questionnaire, such as the wording, order, and range of answers, and the research process, i.e. distribution and collection process [33]. The target was 20 construction workers. 45 practitioners were first contacted via WhatsApp based on convenience sampling. Convenience sampling is a non-probabilistic sampling method that involves respondents who are available and willing to take part [34]. The invitees, who knew the author due to work-related connections, comprised individuals of different job natures, working levels, and locations. The web-based self-administration system “LimeSurvey” was used. The brief introduction and the hyperlink to the survey were included in the WhatsApp message. The participants were also invited to forward it to their friends and colleagues. The pilot survey obtained 23 completed and usable questionnaires with 24 incomplete responses. The preliminary findings and feedback were used to refine the questionnaire.

First, the job nature was adjusted to indicate a more precise background: (1) developer; (2) government; (3) main contractor; (4) sub-contractor; (5) consultant; and (6) others: quasi-government, public utilities. The working level would also be more precise by revising the options to (1) management, (2) project manager, (3) architect, (4) engineer, (5) surveyor, (6) foreman / supervisor, and (7) worker. A respondent stated that some time was required to understand the scale for measuring their attitude “Strictly following rules and procedures is good / bad”. Such scale was therefore revised to (1) strongly disagree to (7) strongly agree to “Strictly following rules and procedures is good” for reducing the difficulty.

The main survey was subsequently conducted. Various construction companies were invited to participate. Before completing the questionnaire, the objectives of the research, as well as the aspects of anonymity and confidentiality, were thoroughly communicated to the participants. The researcher’s colleagues and friends were also invited to forward the invitation email randomly to their acquaintances. The online self-administration platform “LimeSurvey” was created, but ultimately, only a limited number of responses were obtained. The majority of the data was from a hard copy questionnaire.

The population determines the required sample size. The construction sector employs more than 350,000 workers according to the government figure in 2024 [35], so the necessary sample size for a population exceeding 100,000 to achieve precision levels of ±5 %, ±7 % and ±10 %, with a 95 % confidence level and *P* = 0.5, would be 400, 204 and 100, respectively. Furthermore, to ensure reliable estimates for the Structural Equation Modelling (SEM) for analysis (Marsh et al., 1988, as referenced in [35], p. 43), at least 200 participants are required. Considering the various recommendations, the target sample size was confirmed at 300.

The survey resulted in a total of 795 questionnaires. Data screening was performed to confirm the appropriate data for further statistical analyses [37]. As a result, 244 incomplete responses were excluded, leaving 551 complete responses. Subsequently, 46 responses were discarded due to low standard deviation, as suggested by James Gaskin in his YouTube tutorials on data screening, indicating the same answers throughout the questionnaire. Since the literature highlights distinct characteristics, working environment, and safety behaviours of workers, and their immediate supervisory staff (foremen and supervisors), these two categories of respondents were classified. Ultimately, there were 365 valid responses used for data analysis.

### Data collection (interviews)

37 semi-structured interviews were conducted after quantitative data collection and analysis. The interviewees comprise middle managers, supervisors, and workers. The phenomena can be explored from a broader perspective by including some middle managers. Workers can be further classified as Sih-Fus (師傅) (old workers) and young workers. Therefore, there were four groups of respondents. Middle managers refer to professionals such as engineers, project managers, safety officers and managers. Supervisors refer to the frontline staff who lead their subordinates (i.e., workers) in their work. They may have some informal management experience. Since there are no well-established definitions for distinguishing old workers and young workers, old workers are generally called Sih-Fus (師傅). A ten-year cut-off line was adopted in the interview for classification.

The interviews were 30 min for management and supervisors and 20 min for Sih-Fus (師傅) and young workers, respectively. Such length would be sufficient for providing in-depth insight, given the limited concentration of construction workers. Using probing techniques, respondents are allowed to share thoughts on pertinent topics and concepts. Multiple options were provided to aid in brainstorming and to offer a structure for articulating their views. For example, (a) Sih-Fus (師傅) are better; (b) young workers are better; (c) not much difference; and (d) unable to tell, when asking about the comparison of Sih-Fus’ (師傅) safety performance with young workers. After encouraging the participants to voice opinions, the researcher prompted them to expand on their thoughts with justifications and examples. The middle managers were asked an additional question regarding their views on safety management in their organisations (refer to Table 2 for interview questions). The interviews were conducted in Cantonese and audio-recorded. All quotes are literally translated as how the workers say them, and they are non-standard English.

**Table 2:** Interview questions.

Interview Questions
General introduction - What is your role?
- How many years of experience do you have in the industry?
- How many years of employment do you have in this organisation?
View on Sih-Fus (師傅)
- How would you describe a person as Sih-Fus (師傅)?
- Do you describe yourself as Sih-Fus (師傅)? (Yes / no)
- Comparing Sih-Fus (師傅) and young workers’ safety performance
(Sih-Fus (師傅) are better / young workers are better / not much difference / unable to tell)
- How is your daily interaction with workers and supervisors regarding safety?
• With coworkers (Mostly concentrate on your own task / work closely within the same gang / work closely with other Sih-Fus (師傅) [coworkers] / all of the above)
• With supervisors (Follow the instructions from supervisors / discuss with supervisors / all of the above)
Role of Sih-Fus (師傅) in safety
- What is your role [OR Sih-Fus (師傅)’s role] in safety? in shaping workers’ [OR your] attitude to safety?
(Significant / insignificant role)
(Rule compliance / self-discussion / role model / safety engagement / all of the above)
Safety practices
- Do you know how to conduct your activities safely? Why do you think so?
(Yes - better than management and supervisors / yes - under the supervision / no)
- How do you know and share the safety practice?
(Daily briefing or morning assembly / formal workshop / during work / discussion / role model / other means / all of the above)
What is your opinion on the safety rules and procedures of the company or organisation you currently work for?
- Amount (appropriate / too few / too many)
- Level (appropriate / too difficult to understand)
- Applicability to various locations and cultural contexts (applicable / not always applicable)
Do you think that the fatigue issue exists in construction workers? Please provide details. (Yes / no)
Please share your views on the questionnaire survey results and any other factors that affect your compliance with safety rules.
Additional question to middle managers
- Please share your views on safety management in your organisations.

## Analysis and results

### Quantitative analysis and results

The data analysis was conducted using the Statistical Package for the Social Sciences (SPSS) and Analysis of Moment Structures (AMOS). SPSS facilitated the calculation of reliability and the execution of factor analysis, while AMOS was utilised for performing SEM. The reliability and validity of the instrument were checked. Reliability indicates the degree to which the instrument yields consistent and stable results, while validity refers to the extent to which the instrument accurately measures what it intends to measure [38]. Cronbach’s alpha values of all the items were assessed to determine if they exceeded the accepted threshold of 0.7, indicative of acceptable reliability. All items surpassed 0.7 except for safety participation (SP), which scored 0.689. As a result, SP was excluded from further analyses due to its inadequate internal consistency.

Factor analysis aids in comprehending the structure of a collection of variables [39]. There are two primary types of factor analysis: principal components analysis (PCA) and factor analysis. PCA is utilised to condense a large number of variables into a smaller subset, while factor analysis examines the smaller set of factors affecting the observed variables [40]. This study adopted PCA. If there are sound loadings and the communalities are acceptable, no items will be eliminated from further analysis. The factors loading for items Q2 (insufficient time to comply) and P1 (workplace conditions) were below the threshold of 0.4, leading to their exclusion. The three key components of perceived quality found were efficiency (Quality 1), effectiveness (Quality 2), and relevance of rules (Quality 3).

SEM is well known for its advantages that allow the development of complex path models with direct and indirect effects, as well as integrating observed and latent variables within a model [36,41]. A model comprises two elements: the measurement model, which indicates that the observed variables serve as indicators of a latent variable (factor), and the structural model, which outlines the relationship between latent variables and other measured variables [42]. Anderson and Gerbing [43] proposed a two-step procedure for model testing, even though SEM simultaneously assesses direct and indirect relationships among latent and observed variables [36]. Following their suggestion, the Confirmatory Factor Analysis (CFA) model fit and the convergent validity of each construct were first evaluated. Subsequently, the model fit and discriminant validity of the entire measurement model were assessed. After this, the structural model was analysed. For model fit evaluation, two categories of global fit measures were examined to determine if the theoretical model accurately represents the sample data: (1) absolute – assessing the structural model’s capacity to replicate the sample covariance matrix, with criteria such as χ2/degrees of freedom (CMIN/DF) < 3 and Root Mean Squared Error of Approximation (RMSEA), where values below 0.05 indicate good fit, 0.05 to 0.10 moderate fit, and above 0.10 poor fit; and (2) comparative – evaluate the model against a baseline model that performs poorly, using indices such as Comparative Fit Index (CFI) > 0.90 and Tucker-Lewis index (TLI) > 0.90. Values above 0.90 indicate a good model, while values above 0.80 are considered within the acceptable range [44]. If the model does not meet the acceptable fitness indices, modifications will be made.

For testing the measurement model of the individual construct, items should be excluded to enhance the fit if the estimates of an item fall below the necessary threshold of 0.50 [45]. The modification indices (M.I.) for the covariances were identified as error terms that covary within the same factor, and the highest modification indices were prioritised first [45]. The measurement model was not further modified when the modified model presented acceptable fit indices.

**Fig. 2:**
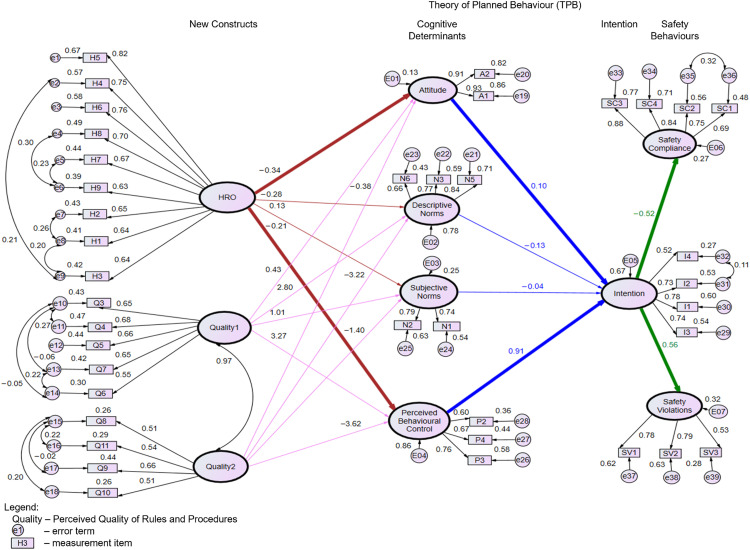
Standardised parameter estimates of modified structural model (Chi-square/df = 2.687, *p*-value = 0.000, TLI = 0.819, CFI = 0.836, RMSEA = 0.068). The significance levels are shown in Table 3, and p ≤ 0.001 is indicated with three asterisks. The confirmed hypotheses are bolded to signify the significant relationships.

The model modified for each construct is summarised as follows: for HRO, error terms e4 covaried with e6 (MI=31.123), e7 covaried with e8 (MI=18.438), e5 covaried with e6 (MI=18.346), e2 covaried with e9 (MI=14.826), and e8 covaried with e9 (MI=11.492). For perceived quality of safety rules and procedures, the factor Quality3 showed that the standardised estimate of Q1 was only 0.21, but Q12 was larger than 1. Therefore, the factor Quality3 was removed. For norms, the standardised estimate of item N4 (supervisors seldom discipline workers who break rules) was 0.48, which was less than the required 0.5 level, so this item was removed to improve the model fit. For both intention and safety compliance, the modified measurement model presented acceptable fit indices after covarying their error terms. The covariates of error terms for individual constructs are represented by arrows with two points in Fig. 2, which presents the outcomes of standardised estimates along with model fit indices for the revised structural model.

The average variance extracted (AVE) among indicators is a summary measure of convergence, which represents how much of the variance in its indicator variables is explained by the construct itself (Fornell & Larcker, 1981, as cited in Hair et al., 2010, p. 687) [46], that is the total of squared standardised factor loadings (square multiple correlations) divided by the number of items:

AVE = (Σ Standardized factor loading^2^)/n

Fornell and Larcker (1981) (as cited in Huang, Wang, Wu, & Wang, 2013, p. 219) [47] suggest that the convergent validity of the construct is still adequate if AVE is less than 0.5 but composite reliability is higher than 0.6. Malhotra and Dash [48] provide a similar argument that AVE is too strict and reliability can be established through composite reliability alone. Each construct achieved this cut-off value, so the convergent validity has been confirmed.

After the acceptable results for the construct fitness indices have been established, the fitness of the complete measurement model with all constructs in the study (proposed framework) and the discriminant validity of the constructs were analysed to confirm each construct is conceptually distinct. The extracted variance should be greater than the squared correlation (i.e. variance shared) between two constructs [49]. Nevertheless, Hair et al. (2010) suggest that this approach of establishing discriminant validity is quite conservative. Some scholars provide the threshold that there is no multicollinearity if the correlations of the constructs are lower than 0.9 (Ozorhon, Arditi, Dikmen, & Birgonul, 2008, p. 366, as cited in Koh, 2010, p. 253 and Memon, 2017, p. 130) [[50], [51]]. In this study, the discriminant validity of constructs has been substantiated by their correlation within the cut-off value of 0.9.

Following the evaluation of the measurement model, it was necessary to assess the effectiveness of the structural model. Similarly, it was essential to evaluate the fitness of the structural model. Convarying was conducted to modify the structural model and improve the fitness indices. After covarying the Quality1 and Quality2 (MI=87.592), the fitness indices of the modified structural model included Chi-square/df=2.687, which is less than 3, a p-value of 0.000, TLI of 0.819, CFI of 0.836, and RMSEA of 0.068. The CMIN/DF demonstrated an acceptable fit, while the TLI, CFI, and RMSEA reached a marginal fit, thus no further modifications were made. By adopting the above steps of reliability and validity testing, the robustness of the method can be substantiated. Fig. 2 illustrates the outcomes of standardised estimates and model fit indices for the revised structural model, while Table 3 presents the path estimates of the model along with their significance levels.

**Table 3:** Unstandardized parameter estimates of modified structural model.

			Estimate	S.E.	C.R.	P (≤ 0.001)
Attitude	<—	HRO	−0.417	.070	−5.916	***
Descriptive Norms	<—	HRO	−0.372	.076	−4.912	***
Subjective Norms	<—	HRO	.137	.068	2.013	.044
Perceived Behavioural Control	<—	HRO	−0.265	.074	−3.600	***
Attitude	<—	Quality1	.563	.372	1.515	.130
Descriptive Norms	<—	Quality1	4.059	1.240	3.274	.001
Subjective Norms	<—	Quality1	1.203	.524	2.297	.022
Perceived Behavioural Control	<—	Quality1	4.419	1.369	3.228	.001
Attitude	<—	Quality2	−0.567	.413	−1.375	.169
Descriptive Norms	<—	Quality2	−5.274	1.407	−3.749	***
Subjective Norms	<—	Quality2	−1.881	.604	−3.115	.002
Perceived Behavioural Control	<—	Quality2	−5.535	1.545	−3.583	***
Intention	<—	Attitude	.098	.050	1.974	.048
Intention	<—	Descriptive Norms	−0.118	.128	−0.922	.356
Intention	<—	Subjective Norms	−0.042	.068	−0.619	.536
Intention	<—	Perceived Behavioural Control	.902	.160	5.625	***
Safety Compliance	<—	Intention	−0.402	.048	−8.345	***
Safety Violations	<—	Intention	.365	.045	8.161	***

The majority of the hypotheses were confirmed. The intention of safety violations was found to significantly impact both safety compliance (H1a) and violations (H1b). Specifically, higher intention would have an impact on lower self-reported safety compliance and higher violations, with a regression weight significance level of 0.001. When intention rose by 1, safety compliance saw a decrease of 0.52, while safety violations experienced an increase of 0.56. Both attitude (H2) and PBC (H4) were found to significantly increase intention, although the influence of attitude (+0.10) was substantially less than that of PBC (+0.91). However, H3 regarding subjective and descriptive norms was not supported, as there were negligible negative effects on the intention of safety violations.

Initially, the substantial negative statistical effects of perceived quality on the three cognitive determinants were not identified, as Quality 1 and Quality 2 demonstrated both significant negative and positive influences on descriptive norms, subjective norms, and PBC. The notable negative statistical impacts of HRO on attitude and PBC were consistent with prior research. Hypotheses H6a and H6c were therefore supported, with regression weights of −0.34 and −0.21, respectively. However, HRO showed a significant positive influence on subjective norms and a negative impact on descriptive norms, leading to the rejection of H6b. These unexpected results were further examined through qualitative interviews.

Regarding the demographic variables, Mann-Whitney tests were conducted to examine if there were significant differences between the mean ranks of the two conditions with different participants in each condition [40]. In comparison, Kruskal-Wallis tests were performed to compare the mean ranks of more than two conditions. The findings indicated that younger subcontractor workers reported significantly lower safety compliance and higher safety violations.

### Qualitative analysis and results

Following quantitative testing of the hypotheses, qualitative interviews were conducted to elicit narrative explanations and explore the phenomenon. Content analysis was performed to analyse the interview scripts. When comparing content analysis and thematic analysis, the purpose of content analysis is “to describe the characteristics of the documents’ content by examining who says what, to whom, and with what effect”.” In contrast, thematic analysis is “an independent qualitative descriptive approach is mainly described as a method of identifying, analysing and reporting patterns (themes) within data” [52]. There are many similarities between content analysis and thematic analysis, but they differ in data quantification [53], as themes tend not to be quantified in thematic analysis [54].

Given the large number of interviews conducted (a total of 37), NVivo (version 12), a qualitative data analysis software, was employed to perform the content analysis. It helps organise and analyse the data more systematically. Kleinheksel et al. [55] suggest four steps: (1) identify units of meaning; (2) label similar units with a code; (3) group codes into a category; and (4) describe related categories with a theme. Recent studies further review the process and recommend a more detailed seven stages: (1) research question; (2) data familiarisation; (3) coding; (4) revising codes; (5) developing the analysis structure; (6) reporting the analysis structure; and (7) interpreting findings with self-reflection at each stage [56]. This recommendation was adopted in this study.

The interview questions form the structure of research questions. The scripts were organised to distinguish the questions and answers, and studied by the author. The initial stage of “noding” – autocoding was carried out by the software according to the questions. Following that, the scripts were examined to code them into smaller subtopics and manual groupings. The analysis structure was then developed and reported. Findings were then interpreted within the TPB and the context of the Hong Kong construction industry. Table 4 shows the coding framework exported from NVivo.

**Table 4:** Coding framework exported from NVivo.

Item	Files	References
Self-introduction		
	31	31
No opinion	2	2
Room for improvement	2	2
Rules okay	7	7
Who are Sih-Fus?	5	5
Definition	1	1
Negative	2	2
Positive	1	1
Sih-Fus versus young workers on safety	22	24
Not different	4	4
Sih Fu better	9	11
Young workers better	10	10
Views on safety in organisations	3	3
Current safety training for Sih-Fus	14	15
Cau-Ga	1	1
No training	10	10
Room for improvement	4	4
Safety engagement of Sih-Fus	16	18
By layers	5	5
Daily communication	6	6
Passive	7	8
Role of Sih-Fus in training young workers	29	32
Contradiction	1	1
Not many young workers	3	3
Not teach	4	4
Role model	1	1
Safety	11	11
Technical things	10	10
Safety promotion (Who is important to promote safety?)	15	17
Everyone	2	2
Foremen	4	4
Main contractor	1	1
Safety officer	6	6
Sih-Fus	2	2
Subcontractor	2	2
Factors affecting safety compliance	31	34
Competitive tendering	2	3
Norms	5	5
PBC	21	23
Safety culture	1	1
Safety rules	6	6
Self-awareness	16	19
Fatigue issue	30	31
Aging	4	4
Effects	7	7
Negative	8	8
Positive	19	19

The background information of the respondents was subsequently imported into NVivo to perform crosstab tests for every question, allowing for the identification of distinguishing patterns, similarities, and differences among all respondents and within each respondent group. The quotations were included with indication of their sources, i.e., from Sih-Fus (師傅) (SF), young workers (YW), supervisors (S), or middle managers (M).

The respondents discussed the questionnaire results and the elements influencing construction workers’ adherence to safety standards, and categorised them into three tiers: (a) micro, (b) *meso*, and (c) macro. Valuable insights were provided for both confirmed hypotheses and those that were refuted. Norms do not exhibit the expected significant positive relationships, which could be attributed to a lack of relational identification in construction settings. Although the results regarding Quality were inconsistent, those interviewed pointed out existing deficiencies in safety rules and procedures, along with a low level of worker engagement in safety management. The rejection of the influence of HRO on subjective norms can be clarified by the HRO concept, which emphasises continuous improvement rather than strict adherence to safety regulations.

In addition to illustrating the factors suggested by the research model, they pinpointed some other institutional contributors, including (a) subcontracting and salary system, and (b) competitive tendering. While these macro elements may not have a direct influence on safety compliance, they could adversely affect both proximal and distal factors. Moreover, the interviews highlighted unique characteristics of the construction industry in Hong Kong, particularly the roles of Sih-Fus (師傅) and *Cau-Gas* (炒家). These two groups are prevalent in this context, and their intriguing interactions offer valuable insights into the current state of the industry. In Hong Kong, seasoned workers with extensive experience are commonly referred to as Sih-Fus (師傅), although there is no definitive description for this term. It is primarily used as a general term, and individuals often categorise based on years of experience. Most respondents indicated that workers with over ten years of experience were labelled as Sih-Fus (師傅). The respondents further illustrated current safety training for Sih-Fus (師傅), and compared Sih-Fus (師傅) with young workers in terms of safety compliance and their role in training young workers. Low safety engagement was revealed among Sih-Fus (師傅). Middle managers also shared views on safety in their organisations.

Regarding the Cau-Ga (炒家) system, it is commonly used for finishing works and scaffolding. Its layout is similar to subcontracting though it operates on a smaller scale. Subcontractors assign specific parts of the work, often segmented by floor or area, to Cau-Gas (炒家), composed of teams of skilled Sih-Fus (師傅). Importantly, these Sih-Fus (師傅) usually work on a daily basis. At the same time, they handle urgent assignments for an extra incentive. This bonus structure is typical for projects with tight deadlines. Consequently, Cau-Gas (炒家) often focus on productivity, which leads them to restrict interactions with less-experienced personnel. The prevalent utilisation of Cau-Gas (炒家) underscores the priority given to work productivity. Furthermore, the respondents noted that everyone involved in construction projects should participate in safety from the outset, covering aspects from the procurement method (competitive tendering) to the overall safety culture within the sector.

## Conclusion and future works

By implementing a mixed-methods approach, this research validates the application of the TPB in this context, which investigates various factors influencing safety violations among construction workers. Specifically, this study has creatively utilised the HRO measurement tool within construction organisations in Hong Kong. The participants discussed various elements that encompass micro, *meso*, and macro factors in detail and highlighted the institutional contributions that explain the poor performance at the work face level and the weakness of management in not being mindful of these issues. The interviews highlighted the unique traits of Sih-Fus (師傅) and Cau-Gas (炒家), which further intensify the issue of safety violations. Regarding practical implications, the relevant interventions can be developed based on the findings of the questionnaire survey and interviews.

### Limitations

Although the reliability and validity of the methodology have been established previously, it is crucial to recognise that all studies have their own constraints. Convenience sampling was adopted in the pilot survey due to limited resources, and snowball sampling was partially adopted in the main survey. There may be biased samples and systematic errors when compared to random sampling [57]. For snowball sampling, although this sampling method is better than convenience sampling, samples become homogeneous over time due to social networks [58]. To relieve this issue, various construction companies were invited to take part in the main survey to provide more reliable and generalisable findings.

The issue of social desirability might be present in the responses given during both surveys and interviews. In this research, the construction workers themselves provided the quantitative data on safety compliance and violations. To mitigate this issue, the researcher highlighted the independence of this study, indicating that it was solely for academic purposes. Respondents were clearly informed about the anonymity and confidentiality measures to alleviate any concerns they might have had. Proper informed consent protocols were adhered to. Additionally, staff from the participating construction companies were asked to refrain from being present with the construction workers while they completed the questionnaire. A data screening process was also conducted to ensure the quality of the data analysis.

Similar to the survey, the ethical issues were clearly explained to them before they signed off the consent form, and the interviews commenced. The confidentiality and anonymity of the interview were emphasised throughout the entire process, as some respondents further clarified this issue when they wished to disclose sensitive information. Appropriate responses were given to encourage them to speak out, allowing them to express their views freely.

### Future works

To improve representativeness in future applications of the method, the research team can cooperate with the worker registration organisation to distribute the questionnaire by adopting a stratified sampling method. In Hong Kong, the Construction Industry Council is responsible for the registration system of construction workers, and they have developed a comprehensive register of all workers. When compared to random sampling, workers of different trades can be selected to complete surveys and interviews, thereby identifying any unique characteristics among various groups of workers. If more resources are available, more sample sizes can be targeted to increase the generalisation of findings further.

The additional factors identified through the interviews, combined with the results of safety behaviours such as injury rates and absenteeism, can be integrated into the research model for further analysis, providing objective measurements and directions for future research. When more time is allowed in future research, a longitudinal study can be conducted to examine the changes in safety behaviours and the impact of factors over time and throughout construction stages, since the dynamics would be very different from project commencement, peak period, and completion. Given the intricate nature of safety violations, it is proposed that an ethnographic investigation exploring their social interactions in everyday tasks.

This study incorporated safety participation into the model; however, the low reliability of the scale was identified, so it was excluded from further analyses. This may result from different perspectives represented in each of the three questions. It is suggested that safety participation can be examined in future research to provide deeper insights into safety behaviours. The participants in the interviews highlighted concerns about the low safety engagement of workers. Additionally, safety engagement can be analysed to uncover various aspects of safety behaviours going forward.

## Ethics statements

All participants provided informed consent in written form before participating in the questionnaire survey and interview. Before giving informed consent, all participants were informed about the purpose of the study, the voluntary nature of their participation, the confidentiality of their responses, and their right to withdraw at any time without consequences.

## CRediT author statement

**Wing Chi Tsang:** Conceptualisation, Methodology, Validity tests, Writing – original draft preparation. **Shoeb Ahmed Memon:** Writing – Reviewing and Editing. **Steve Rowlinson:** Supervision.

## Declaration of competing interest

The authors declare that they have no known competing financial interests or personal relationships that could have appeared to influence the work reported in this paper.

## Data Availability

The data that has been used is confidential.
